# Presentation of Study Results: The Authors’ Responsibility

**DOI:** 10.1289/ehp.1205556

**Published:** 2012-08-31

**Authors:** Igor Burstyn, Brian Lee, Nicole B. Gidaya, Michael Yudell

**Affiliations:** Drexel University, Philadelphia, Pennsylvania, E-mail: igor.burstyn@drexel.edu

We read with interest the article by Kalkbrenner et al. (2012) in which they explored maternal smoking during pregnancy as a risk factor for autism spectrum disorders (ASD). We believe that the following shortcomings of the study did not allow an evaluation of the results and therefore that the paper provides little evidence to judge whether data suggest a “link.”

The findings of Kalkbrenner et al. (2012) regarding “higher-functioning” ASD include three null associations and one association in the smallest subgroup of 375 cases (ASD-not otherwise specified; ASD-NOS) that was “statistically significant” only in sensitivity analysis. Therefore, we question their interpretation of the data when an effect was suggested in only one of the four tests of the same hypothesis. Furthermore, ASD-NOS is a difficult diagnostic subtype to understand because it includes, as the authors noted, a heterogeneous mixture of diagnoses.

Although socioeconomic status (SES) is a well-known correlate of both smoking and ASD, the authors used only maternal education to control for SES; thus, residual confounding from other aspects of SES is likely (King and Bearman 2011; Rai et al. 2012).

Kalkbrenner et al. (2012) did not appropriately control for confounders, and this affected sensitivity analysis central to their conclusions. In their sensitivity analysis for outcome misclassification, they did not correct for covariates, thus basing all of their interpretations on results that were contaminated by confounding. They could have used Monte Carlo methods (Bodnar et al. 2010) to adjust for confounding while accounting for outcome misclassification, obtaining confidence intervals that account for random simulation error, but they did not do this. Thus, the reported confidence intervals for the sensitivity analyses are likely to be too narrow.

Kalkbrenner et al. (2012) did not quantitatively assess the impact of exposure misclassification. The quoted 0.8 concordance of smoking data on birth certificates with the medical record means that smoking exposures of > 125,000 persons in the sample were expected to be incorrectly classified. Sensitivity of maternal smoking on U.S. birth certificates is likely to be only 0.5 (Kharrazi et al. 1999). Epidemiologists ignore measurement error at great peril (Jurek et al. 2006) while correction procedures exist (MacLehose and Gustafson 2012).

Finally, we would like to point out the difficulties of this article in communicating scientific results to the general public. Because, as Kalkenbrenner stated, “the study doesn’t say for certain that smoking is a risk factor for autism” (UWM News 2012), then it is the author’s responsibility to more carefully report to the media what the study actually does say. It is easy to blame journalists for the sensational findings that have been reported about this study (e.g., Goodwin 2012). However, given the historic legacy of blaming parents, particularly mothers, for their child’s diagnosis, we would better serve the communities for whom we do this research if we developed standard practices for reporting preliminary findings in ASD risk factor research. One suggestion would be to report these findings without discussion in media (e.g., Palmer 2011) and scholarly publications, as was done by Adam et al. (2011), who produced experimental data demonstrating that the speed of light was exceeded:

Despite the large significance of the measurement reported here and the robustness of the analysis [*p* << 0.00006%], the potentially great impact of the result motivates the continuation of our studies in order to investigate possible still unknown systematic effects that could explain the observed anomaly. We deliberately do not attempt any theoretical or phenomenological interpretation of the results.

We encourage caution when promoting findings of “potentially great impact” on public health that are of a preliminary nature and are not ready to be even interpreted.
